# Skin Lesions as Signs of Neuroenhancement in Sport

**DOI:** 10.3390/brainsci15030315

**Published:** 2025-03-17

**Authors:** Sorana-Cristiana Popescu, Roman Popescu, Vlad Voiculescu, Carolina Negrei

**Affiliations:** 1 Department of Toxicology, Faculty of Pharmacy, “Carol Davila” University of Medicine and Pharmacy, 020945 Bucharest, Romania; sorana-cristiana.pruna@drd.umfcd.ro (S.-C.P.);; 2 Department of Orthopaedics, “Carol Davila” University of Medicine and Pharmacy, Rectorate—Dionisie Lupu Street, No. 37, District 1, 020021 Bucharest, Romania; 3 Elias University Emergency Hospital—Marasti Boulevard, No. 17, District 1, 011461 Bucharest, Romania

**Keywords:** neuroenhancement, skin lesions, cognitive enhancers, rivastigmine, donepezil, transcranial direct electrical stimulation, neurodoping, anti-doping

## Abstract

Background: Neuroenhancement in sports, through pharmacological and non-pharmacological methods, is a complex and highly debated topic with no definitive regulatory framework established by the World Anti-Doping Agency (WADA). The hypothesis that dermatological changes could serve as observable biomarkers for neurodoping introduces a novel and promising approach to detecting and understanding the physiological impacts of cognitive enhancers in athletes. As neurodoping methods become increasingly sophisticated, developing objective, reliable, and non-invasive detection strategies is imperative. Utilizing dermatological signs as a diagnostic tool for internal neurophysiological changes could offer critical insights into the safety, fairness, and ethical considerations of cognitive enhancement in competitive sports. A systematic correlation between skin manifestations, the timeline of neurodoping practices, and the intensity of cognitive enhancement methods could provide healthcare professionals valuable tools for monitoring athletes’ health and ensuring strict compliance with anti-doping regulations. Methods: Due to the limited body of research on this topic, a systematic review of the literature was conducted, spanning from 2010 to 31 December 2024, using databases such as PubMed, Science Direct, and Google Scholar. This study followed the 2020 PRISMA guidelines and included English-language articles published within the specified period, focusing on skin lesions as adverse reactions to pharmacological and non-pharmacological neuroenhancement methods. The research employed targeted keywords, including “skin lesions AND rivastigmine”, “skin lesions AND galantamine”, “skin lesions AND donepezil”, “skin lesions AND memantine”, and “skin lesions AND transcranial direct electrical stimulation”. Given the scarcity of studies directly addressing neurodoping in sports, the search criteria were broadened to include skin reactions associated with cognitive enhancers and brain stimulation. Eighteen relevant articles were identified and analyzed. Results: The review identified rivastigmine patches as the most used pharmacological method for neuroenhancement, with pruritic (itchy) skin lesions as a frequent adverse effect. Donepezil was associated with fewer and primarily non-pruritic skin reactions. Among non-pharmacological methods, transcranial direct current stimulation (tDCS) was notably linked to skin burns, primarily due to inadequate electrode–skin contact, prolonged exposure, or excessive current intensity. These findings suggest that specific dermatological manifestations could serve as potential indicators of neurodoping practices in athletes. Conclusions: Although specific neuroenhancement methods demonstrate distinctive dermatological side effects that might signal neurodoping, the current lack of robust clinical data involving athletes limits the ability to draw definitive conclusions. Athletes who engage in neurodoping without medical supervision are at an elevated risk of adverse dermatological and systemic reactions. Skin lesions, therefore, could represent a valuable early diagnostic marker for the inappropriate use or overuse of cognitive-enhancing drugs or neuromodulation therapies. The findings emphasize the need for focused clinical research to establish validated dermatological criteria for detecting neurodoping. This research could contribute significantly to the ongoing neuroethical discourse regarding the legitimacy and safety of cognitive enhancement in sports.

## 1. Introduction

Neuroenhancement involves boosting cognitive capacity by influencing how healthy brains adapt to various situations using pharmacological and non-pharmacological methods. According to DSM-5 [1], six key cognitive domains are critical to understanding neuroenhancement: complex attention, executive function, learning and memory, language, perceptual-motor function, and social cognition. Complex attention involves focusing on relevant information while filtering out distractions. Executive function relates to planning, organizing, and sequencing tasks. Learning and memory pertain to storing and retaining information for short- and long-term access. Language is a defining human trait that facilitates oral and written communication. Perceptual-motor control is the ability to coordinate body movements and navigate the environment. Social cognition influences how we process and respond to our own and others’ emotions, guiding behavior and social interactions. Understanding these domains helps clarify how neuroenhancement targets specific cognitive functions and how improvements may vary depending on the approach used.

Jangwan et al. [2] outlined that the history of neuroenhancement began in 1780 with Luigi Galvani’s discovery that electrical stimulation could trigger muscle movements in dead frogs. In 2020, the Stentrode brain–computer interface enabled two patients to perform daily activities such as shopping and messaging using a Surface Book 2 running Windows 10, marking a milestone as the first brain–computer interface implanted via blood vessels without open-brain surgery.

Various behavioral, physical, and pharmacological strategies can target each domain to enhance cognition. Behavioral methods, non-invasive, cost-effective, and healthy, include aerobic exercises, sleep optimization, meditation, yoga, and dietary adjustments.

This article focuses on neuroenhancement in sports through pharmacological and non-pharmacological (physical) methods, which remains highly debated and lacks clear regulatory guidelines [3,4].

Pharmacological methods involve using substances that enhance cognitive performance but are not prohibited by the World Anti-Doping Agency (WADA). These substances, known as “nootropics”, include Alzheimer’s medications such as Donepezil, Memantine, Galantamine, and Rivastigmine, which primarily improve learning and memory.

Non-pharmacological (physical) methods include both invasive techniques (e.g., Deep Brain Stimulation) and non-invasive strategies (e.g., Transcranial Direct Current Stimulation, Transcranial Magnetic Stimulation, Transcutaneous Vagus Nerve Stimulation, and neurofeedback) [2].

Originally developed to aid recovery from neurodegenerative and vascular conditions such as Alzheimer’s, Parkinson’s, and stroke, these cognitive enhancement methods have gained popularity in sports. The intense pressure on elite athletes to continually exceed performance limits, including financial and competitive stresses, has increased interest in neuroenhancement [5]. While these interventions’ neurological and performance outcomes are well-studied, their effects on skin health remain largely unexplored.

Given that the skin is the body’s largest organ and the primary interface with the external environment, our research introduces a novel perspective: the potential for skin lesions to indicate the use of pharmacological or physical neuroenhancement methods. This is particularly significant since WADA does not currently prohibit these methods.

Since detection kits for cognitive enhancers are costly and the substances used are not banned, the clinical examination of skin lesions could provide a practical and accessible method for sports doctors to identify potential neurodoping cases. Our research bridges cognitive enhancement, dermatology, and sports medicine, focusing on dermatological manifestations of neuroenhancement strategies, including Alzheimer’s medications and neuromodulation techniques like Transcranial Magnetic Stimulation (TMS).

As neurodoping techniques evolve, objective and non-invasive detection methods become critical. Skin changes as potential biomarkers for neurodoping could offer healthcare professionals valuable tools for monitoring athletes’ health and ensuring compliance with anti-doping regulations. Correlating dermatological signs with the intensity and timeline of neurodoping practices may lead to improved safety, ethical standards, and regulatory practices in competitive sports.

## 2. Materials and Methods

### Selection Procedure and Inclusion Criteria

This study was developed as a systematic review to summarize the available research on skin lesions associated with pharmacological and non-pharmacological methods of enhancing cognitive performance. The systematic review was conducted following the 2020 Preferred Reporting Items for Systematic Reviews and Meta-Analyses (PRISMA) criteria [6], although it was not registered.

Scientific databases, including PubMed, Science Direct, and Google Scholar, were searched using specific keywords and Boolean operators: “skin lesions AND rivastigmine”, “skin lesions AND galantamine”, “skin lesions AND donepezil”, “skin lesions AND memantine”, and “skin lesions AND transcranial direct electrical stimulation”.

Inclusion Criteria:

1. Articles published in English.
2. Publications from 2010 to 31 December 2024.
3. Studies focused on skin lesions as adverse reactions to pharmacological and non-pharmacological neuroenhancement methods.

Exclusion Criteria:

We excluded studies that, despite meeting the inclusion criteria, were eliminated for specific reasons:

1. Articles not published in English.
2. Studies were published before 2010.
3. Review articles, systematic reviews, or meta-analyses.
4. Studies where skin lesions were not the primary focus of adverse reactions, explicitly excluding articles that predominantly reported cardiological, neurological, or gastroenterological side effects. The rationale for this exclusion was to maintain a focused scope on dermatological signs, which may serve as subtle yet valuable indicators of neurodoping in athletes.

**Figure 1:**
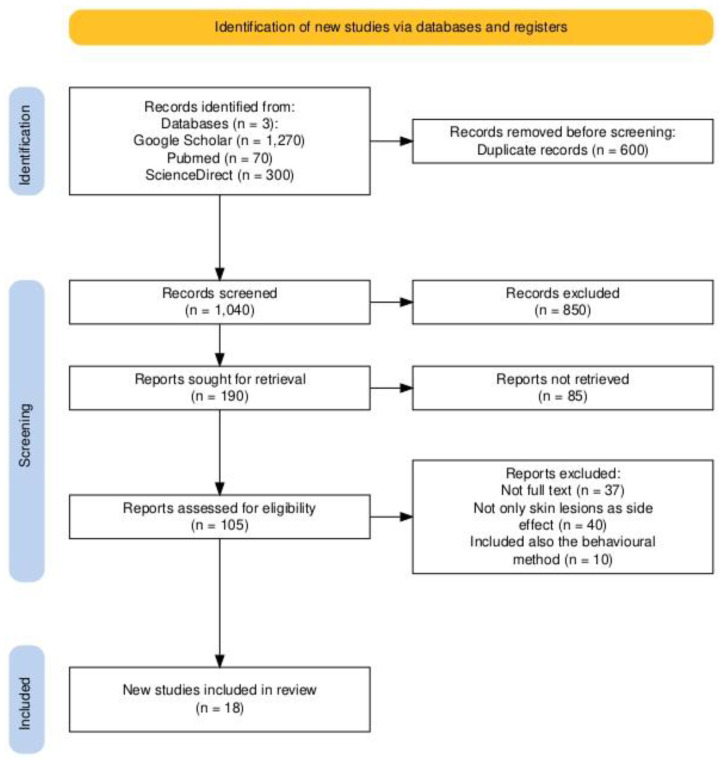
PRISMA 2020 flowchart illustrating the study selection process.

Due to the lack of studies directly addressing skin lesions in athletes using neuro-enhancement medication, we broadened our search to include research on skin lesions associated with the selected neuro-enhancement medications and non-invasive, non-pharmacological procedures. The selection process adhered strictly to PRISMA criteria, which we have visually summarized in an organizational flowchart (Figure 1) [6].

**Data Extraction and Analysis**: Two reviewers (RP and VV) independently conducted the selection procedure, data extraction, and analysis according to predefined inclusion criteria. CN’s coordination resolved disagreements between reviewers, ensuring a consensus was reached.

**Study Selection Process:** The initial database search yielded 1640 articles. After removing duplicates, 1040 articles remained. Applying the exclusion criteria further reduced the pool to 105 articles. From these, eighty-five were unsuitable, leaving eighteen articles that met all inclusion criteria and were included in the final analysis. The eighty-seven excluded articles either discussed behavioral methods, included multiple side effects beyond skin lesions, or lacked full-text availability.

The detailed selection procedure is illustrated in Figure 1.

## 3. Results

The final selection included 18 articles categorized as follows: (Section 3.1) studies on skin lesions associated with Rivastigmine use; (Section 3.2) studies on skin lesions linked to Memantine use; (Section 3.3) studies examining skin lesions related to Donepezil use; and (Section 3.4) studies investigating skin lesions following Transcranial Direct Electrical Stimulation. The results are summarized in Table 1 and Table 2.

### 3.1. Studies Investigating Skin Lesions Associated with the Use of Rivastigmine

Rivastigmine is a treatment used to manage neurodegenerative pathologies, particularly dementia and its associated symptoms. It belongs to the cholinesterase inhibitor class of drugs. The brain contains two cholinesterase enzymes, acetylcholinesterase and butyrylcholinesterase, which hydrolyze acetylcholine. Acetylcholine is a critical neurotransmitter involved in superior cognitive functions such as complex attention, learning, memory, and voluntary muscle movement. As people age, the activity of these enzymes increases, reducing acetylcholine levels and leading to cognitive and muscular dysfunctions [25].

Navarro-Triviño and Ruiz-Villaverde [7] reported a case of an 84-year-old male treated with a 9.5 mg/24 h transdermal Rivastigmine patch. After three weeks of treatment, he developed intense itching and eczematous plaques where the patches were applied. The authors concluded that the pruritic lesions were due not to rivastigmine but to the patch adhesive compound, specifically 2-Ethylhexyl acrylate.

Grieco et al. [10] presented a case involving a 75-year-old male who developed itchy rashes on his trunk and proximal upper limbs after 15 days of treatment with Rivastigmine 9.5 mg/24 h. Similarly, Golüke et al. [8] described a 74-year-old male initially treated with a 4.6 mg rivastigmine patch. After three months without adverse reactions, the dose was increased to 9.5 mg/24 h. A year later, the patient developed pruritus and an allergic urticaria rash on his left flank, attributed to rivastigmine use rather than the adhesive compound.

Imbernón-Moya et al. [9] reported a rare case of hypertrichosis. An 80-year-old male developed symmetrical hypertrichosis on the dorsal sides of his forearms after about a month of oral rivastigmine treatment at a dose of 3 mg/12 h. Another case report by Makris et al. [11] involved an 85-year-old female who experienced a delayed hypersensitivity reaction, including pruritus and maculopapular erythematous lesions. This reaction was triggered by rivastigmine patches (initially 4.6 mg for 8 weeks, then increased to 9.5 mg/24 h for 15 days) and oral rivastigmine (6 mg oral solution/day for only two days).

Chouchana et al. [15] described a 79-year-old male who developed a maculopapular eruption using a 9.5 mg/24 h rivastigmine patch (increased from 4.6 mg/24 h). Switching to oral rivastigmine (1.5 mg twice a day) worsened his skin lesions. Allain-Veyrac et al. [12] reported an 88-year-old male treated with 1.5 mg rivastigmine twice daily, who developed pruritus and an erythematous macular rash in the axillary and groin areas a year later.

Alva et al. [13] conducted a study comparing the occurrence of skin lesions among patients using different doses of rivastigmine patches (4.6 mg, 9.5 mg, and 13.3 mg per 24 h). They examined how the dose and duration of treatment influenced the development of skin reactions. Naharci and Tasci [14] documented a case of an 84-year-old female treated with a 4.6 mg/24 h rivastigmine patch for one month. She developed angioedema, which presented swollen lips, tongue, and lower eyelids.

### 3.2. Studies Investigating Skin Lesions Associated with the Use of Memantine

Memantine is a low-affinity, voltage-dependent, uncompetitive antagonist of NMDA receptors [26]. NMDA receptors are unique because they require the simultaneous binding of two distinct agonists (glutamate and glycine/D-serine) for activation. In the central nervous system, NMDA receptor activation primarily depends on the synaptic release of glutamate. In neurodegenerative diseases like Alzheimer’s and Parkinson’s, NMDA receptor-mediated excitotoxicity is thought to cause neuronal death, contributing to brain atrophy and dementia [27]. Unlike high-affinity NMDA antagonists such as ketamine, which can induce psychosis-like symptoms, Memantine is a low-affinity NMDA blocker that decreases amyloid-beta levels and enhances cognitive function [26,27].

Mancano [16] reported an 89-year-old male treated with Memantine and Donepezil for two months who developed erythema and papules on his extremities and chest. The symptoms resolved after discontinuing Memantine. Saito et al. [17] presented a similar case of an 89-year-old male also treated with Memantine and Donepezil, who developed an erythematous eruption on the trunk and extremities, primarily in flexures, after two months of treatment. The symptoms also resolved upon the discontinuation of Memantine.

### 3.3. Studies Investigating Skin Lesions Associated with Using Donepezil

Donepezil is a second-generation cholinesterase inhibitor, like Rivastigmine, that reversibly inhibits acetylcholinesterase, enhancing cholinergic transmission [28]. Sabbagh et al. [18] conducted a randomized study with 256 participants who received either a placebo or a donepezil transdermal delivery system. After three weeks, a minimal rash was observed among the treatment group. Hussian et al. [19] described an 84-year-old female who developed a rash two weeks after starting Donepezil, which cleared up once the medication was discontinued.

### 3.4. Studies Investigating Skin Lesions Associated with Transcranial Direct Electrical Stimulation

Transcranial direct electrical stimulation (tDCS) is a non-invasive brain stimulation method developed in the 1950s and introduced for psychiatric treatment in the 2000s. It modulates brain activity and excitability by applying weak direct currents to neuronal membranes [29]. The device consists of an anode, a cathode, connecting wires, and a current generator that delivers a constant electrical current of up to 2 mA [30,31].

The effect of tDCS depends on the intensity of the stimulus. It can either increase cortical excitability via the anode or decrease it via the cathode, which is important for modulating motor learning. Kortteenniemi et al. [20] reported two female patients, aged 18 and 19, who participated in tDCS experimental studies with a 1.5 mA current for 15 min. Two days later, the younger patient developed a lesion on the back of her wrist, and the older patient had a non-itchy lesion on the palmar side of her wrist.

Lu and Lam [21] described three patients (one male, two females, average age of 69) who experienced mild erythema and skin burning at the cathode site after the fourth or fifth tDCS session. The authors suggested that skin resistance might play a role in these reactions. Maas et al. [23] reported skin burns under the cathode terminal in a 47-year-old male, occurring after his tenth tDCS session. The burns appeared below the electrode’s corners, which were placed on a tattoo.

Frank et al. [22] presented three cases regarding skin lesions at the anode site after the fourth tDCS session with a 1.5 mA current. Wang et al. [24] described a 25-year-old male who sustained a skin burn after a single tDCS session using an intensity higher than 2 mA for 26 min. The higher current and prolonged exposure likely contributed to the adverse skin reaction

## 4. Discussion

The increasing use of neuroenhancement drugs and methods is a growing yet under-researched issue, particularly in the realm of competitive sports. Cognitive enhancement presents a new challenge for the World Anti-Doping Agency (WADA) [5]. While cognitive drugs and non-invasive methods such as transcranial direct current stimulation (tDCS) were initially developed to treat neurodegenerative diseases (e.g., Alzheimer’s) and psychiatric disorders, their non-medical use for enhancing cognitive performance constitutes neurodoping. These methods enhance cognitive functions like attention, memory, learning, and voluntary movement by chemically or electrically stimulating neurotransmitters.

This article aims to raise awareness about the non-medical self-administration of neuroenhancement methods as covert doping strategies in sports, especially since their use is challenging to detect. Although WADA’s prohibited list includes many cognitive performance-enhancing drugs and methods, the true extent of neuroenhancement practices in sports remains unknown mainly [5,32].

Neuroenhancement’s ethical and regulatory implications in sports are critical to this discourse. Neurodoping, by enhancing cognitive functions such as focus, decision-making, and reaction times through pharmacological or non-pharmacological means, raises substantial concerns about fairness, athlete health, and the integrity of competition. From a neuroethical perspective, neurodoping challenges core principles of sportsmanship by creating inequitable conditions among athletes. Unlike traditional forms of physical doping, which enhance physiological performance, neurodoping manipulates mental processes, potentially offering an advantage that is not attributable to natural talent or disciplined training. This discrepancy undermines the concept of meritocracy in sports, where success should be a function of skill, hard work, and strategic preparation. Moreover, neurodoping risks redefining societal achievement standards, shifting value away from perseverance and innate ability toward artificially enhanced performance. The health implications of neurodoping extend beyond dermatological side effects. Pharmacological agents like rivastigmine and donepezil and neuromodulation techniques like transcranial direct current stimulation (tDCS) carry potential risks.

These include acute adverse effects such as cardiovascular, neurological, and psychiatric reactions, and chronic consequences like altered brain plasticity and cognitive dependency. The off-label use of these substances without medical oversight can amplify these risks, particularly among athletes who may already be under physical and mental strain. Regulatory bodies such as the World Anti-Doping Agency (WADA) are crucial in establishing clear guidelines and regulatory frameworks to address neurodoping. Currently, the WADA has not explicitly classified specific neuro enhancement methods as prohibited, highlighting a regulatory gap. The evolving landscape of cognitive enhancement technologies necessitates a proactive approach from regulatory institutions, including the periodic review of prohibited lists, the development of new testing methodologies, and the implementation of educational programs for athletes and sports professionals. To support these efforts, our study emphasizes the potential role of dermatological signs as non-invasive biomarkers for neurodoping. By providing sports physicians with practical guidelines for identifying suspicious skin lesions, we aim to contribute to early detection strategies that align with ethical and regulatory standards. Furthermore, our research advocates an informed and balanced approach to neurodoping, encouraging the sports community to engage in ongoing research, transparent discussions, and developing evidence-based policies that prioritize athlete health, safety, and fairness [33].

Current methods for detecting brain stimulation in athletes, such as magnetic resonance spectroscopy (MRS), are costly and logistically challenging, as they require testing both before and after brain stimulation [34]. Consequently, examining cutaneous lesions—potential side effects of neuroenhancement methods—may offer a more practical approach to detecting neurodoping. Since existing literature does not explicitly link athletes’ skin lesions to neuroenhancement practices, this study aimed to correlate known dermatological side effects with these methods.

According to Bahrani et al. [35], medications for neurological diseases are associated with various cutaneous adverse effects. Donepezil, for example, may cause pruritus, mild rash, and purpuric rash on the trunk and extremities. Rivastigmine has been linked to allergic and irritant contact dermatitis or “Baboon syndrome” (a type of systemic dermatitis), whereas Memantine is not known to cause skin reactions. Additionally, the literature describes skin lesions linked to WADA-prohibited doping substances, including rosacea and toxic epidermal necrolysis (from amphetamines), alopecia, hirsutism in women, acne, and rosacea (from anabolic steroids), as well as exanthema and linear IgA dermatosis (from diuretics) [36].

Our study found that rivastigmine patches, particularly the 9.5 mg patch administered over 24 h (equivalent to 12 mg/day orally), frequently cause itchy skin lesions. Moderate to severe erythema occurred in 7.6% of patients, while 2.3% experienced pruritus. Women, who are more prone to allergic dermatitis and excessive sweating, face increased risks of skin irritation when using these patches [37,38]. Rivastigmine plasma concentrations gradually increase post-patch administration, peaking at around eight hours regardless of dose, with a half-life of 3.2 to 3.9 h. Skin lesions may persist for up to 48 h after patch removal [39].

Regarding Donepezil, our research found predominantly non-itching skin lesions. Higher doses of Donepezil correlate with increased adverse reactions, with pallor being the primary skin-related side effect. The co-administration of N-acetylcysteine (2400 mg/kg) has been suggested to reduce pallor significantly by days 8–12 of treatment [40]. Unlike rivastigmine, Donepezil has a longer half-life (63 h) and a tmax of 3–4 h, enhancing the detectability of its metabolites [41].

Two case reports indicated that Memantine could cause erythematous lesions. Memantine’s half-life ranges from 60 to 100 h, with a tmax of 6–8 h post-administration. Primarily excreted renally as unchanged drug (75–90%) or hydroxylated metabolites, Memantine’s metabolites are easier to detect than Donepezil [42]. Although Memantine has fewer dermatological side effects, its potential for neurodoping should not be overlooked due to its effectiveness in treating neurodegenerative diseases.

In the case of electrical brain stimulation, the most common dermatological effect is skin burns, often in the temple area. Demonstrating the use of this method as neurodoping is particularly challenging, and skin lesions remain the most visible indication of its use. Unlike pharmacological methods, which are often designed for specific therapeutic purposes, electrical brain stimulation may also be used legitimately for conditions such as migraines, depression, or anxiety, complicating the task of proving neurodoping. However, neuroenhancement may be suspected if an athlete lacks a medical history for such conditions but presents with characteristic skin lesions. A comprehensive review of over 33,200 tDCS sessions involving more than 1000 subjects reported skin burns due to poor electrode-skin contact or irritation [43].

We propose preliminary guidelines for sports physicians to address the challenge of detecting neurodoping through dermatological signs. Clinicians should be vigilant for specific skin manifestations, including the sudden onset of atypical rashes or lesions without a clear etiology, persistent skin eruptions resistant to standard treatments, delayed wound healing, and abnormal scarring. These cutaneous signs could warrant further investigation, primarily when unexplained by known dermatological conditions. A multifaceted clinical approach is recommended, combining thorough skin examinations during routine athlete assessments with advanced imaging technologies. Dermoscopy, optical coherence tomography, and reflectance confocal microscopy can provide non-invasive insights into skin changes potentially associated with neurodoping. Skin biopsies could be considered to analyze histopathological changes in more complex cases. Emerging technologies, such as sweat and sebum analysis, offer promising avenues for detecting neuroenhancement substances or their metabolites directly from skin secretions. Additionally, wearable devices that monitor skin physiology—such as temperature, hydration, and electrical conductance—could detect physiological anomalies linked to neurodoping practices. Implementing these strategies in sports medicine could enhance the early detection and management of neurodoping, contributing to fairer and safer competitive environments [44].

## 5. Limitations

The present review has several limitations. Firstly, despite identifying skin side effects associated with both pharmacological and non-pharmacological neuroenhancement methods, establishing a definitive correlation between an athlete’s skin lesions and cases of neurodoping remains challenging. This difficulty arises from the nonspecific nature of dermatological manifestations, which may be attributed to various causes, including environmental factors, existing dermatological conditions, or mechanical injuries related to sports activities.

Secondly, the absence of clinical studies focusing specifically on athletes using neuroenhancement methods for performance enhancement limits the ability to draw robust conclusions. While certain substances and techniques listed as prohibited by WADA have well-established performance-enhancing effects, the same level of evidence does not yet exist for neurodoping methods. This gap in research contributes to the ongoing debate surrounding these methods and WADA’s undecided stance on their regulation. Additionally, the variability in skin reactions due to individual factors such as skin type, age, and pre-existing skin conditions adds another layer of complexity to diagnosis. Furthermore, practical challenges in real-world settings, such as differentiating between legitimate therapeutic use and illicit enhancement practices, complicate the implementation of anti-neurodoping measures.

Nevertheless, this review’s greatest strength lies in its synthesis of the latest data on neurodoping. It highlights a novel and less explored approach to detecting potential neurodoping in athletes by focusing on readily observable and visible signs—cutaneous side effects. These insights could pave the way for developing new diagnostic tools and protocols, offering sports physicians a practical method for early detection and intervention in suspected cases of neurodoping.

## 6. Conclusions

Our findings indicate that specific pharmacological neuroenhancement methods, such as rivastigmine patches, are more likely to produce dermatological side effects, making them potential indicators of neurodoping. Similarly, non-pharmacological techniques like transcranial direct current stimulation (tDCS) may lead to detectable skin burns, often associated with improper use of electrodes. However, without robust clinical studies involving athletes, establishing a definitive link between dermatological signs and neurodoping remains challenging. The convergence of neurodoping with treatments for neurological conditions, such as Alzheimer’s disease, and with techniques like tDCS, presents a distinct and underexplored risk profile. Athletes who engage in neurodoping without medical oversight face increased risks of adverse reactions, including dermatological manifestations. Consequently, skin lesions could be an early diagnostic marker for the misuse or inappropriate dosing of cognitive-enhancing substances or neuromodulation therapies.

## 7. Future Lines of Research

Considering the scarcity of literature connecting neuroenhancement and neurodoping in athletes, our study opens new avenues for research into how cognitive enhancers, known for their impact on physical and mental performance, might be misused in competitive sports. Future research could focus on developing specific detection methods, including targeted diagnostic kits for neuroenhancement substances, like the existing anti-doping technologies employed by the WADA. Additionally, our findings contribute to the broader neuroethical discourse surrounding fairness, safety, and the integrity of sports. Future studies should prioritize longitudinal and experimental research involving athletes to substantiate the proposed link between skin lesions and neurodoping practices, ultimately supporting the creation of objective clinical guidelines for sports physicians.

## Figures and Tables

**Table 1:** Summary of selected studies.

Article	Article Design	Year	Group (Male/Female, Age)	Type of Skin Lesion and Localization	Time Elapsed Since the Onset of Skins Lesions	Pharmaceutical /Non-Pharmaceutical Method
1. Navarro-Triviño F.J.N and Ruiz-Villaverde R. [7]	Case report	2020	Male, 84	Multiple round erythematous–edematous lesions on the chest	3 months	Rivastigmine patch 9.5 mg/24 h
2. Golüke et al. [8]	Case report	2014	Male, 74	Pruritus and allergic rash on left flank	1 year	Rivastigmine patch 9.5 mg/24 h
3. Imbernón-Moya et al. [9]	Case report	2016	Male, 80	Progressive asymptomatic hair growth on forearms	3 months	Rivastigmine oral −3 mg/12 h
4. Grieco et al. [10]	Case report	2011	Male, 75	Erythematous–edematous lesions on chest and arms	15 days	Rivastigmine patch 9.5 mg/24 h
5. Makris et al. [11]	Case report	2010	Female, 85	Maculopapular itchy rash, hypersensitivity	8 weeks	Rivastigmine patch 4.6 mg/24 h
				Maculopapular eruption on the anterior and posterior chest, arms with progressive extension	The second day	Rivastigmine oral solution 6 mg/24 h
6. Allain-Veyrac et al. [12]	Case report	2011	Male, 88	Itchy, symmetrical, pure-purple erythematous rashes in the axilla and groin (SDRIFE or else named Baboon Syndrome)	4 months	Rivastigmine 1.5 mg × 2/24 h
7. Alva et al. [13]	Original article	2015	ACTION TRIAL Male and Female > 50 years old	Erythema on application site reaction (12.5%) Site dermatitis (8.4%)	24 weeks	Rivastigmine patch 13.3 mg/24 versus 4.6 mg/24 h
			OPTIMA TRIAL Male and Female > 50 years old	Erythema on application site reaction	72–96 weeks	Rivastigmine patch 9.5 mg/24 h versus 13.3 mg/24 h
			IOL (initial open-labeled)	11.6% IOL	24–48 weeks	
			DB (randomized double-dummy)	6.0% DB	48 weeks	2 patches—one placebo and one active
				Site dermatitis 0.9% IOL 0% DB		
8. Naharci and Tasci [14]	Case report	2018	Female, 84	Lower lip and tongue swollen Mild swelling of upper and lower eyelids	1 month and 3 days with the 9.5 mg patch	Rivastigmine patch 4.6 mg/24 h to 9.5 mg/24 h
9. Chouchana et al. [15]	Case report	2024	Male, 79	Pruritic erythematous maculopapular eruption at patch application site	3 months 1 month	Rivastigmine 4.6 mg/24 h Rivastigmine 9.5 mg/24 h
				Spread of the rash over 70% of the body surface, including arms, legs, chest and abdomen	2 weeks	Rivastigmine oral 1.5 mg × 2/24 h
10. Mancano [16]	Case report	2018	Male, 79	Erythematous eruptions and papules on trunk and extremities	2 months	Memantine
11. Saito et al. [17]	Case report	2017	Male, 89	Erythematous eruptions on chest and extremities, mainly on flexures	2 months	Memantine
12. Sabbagh et al. [18]	Original article	2023	256 participants (male and female) > 40 years old	Mild skin irritation at the application site	Day 22 (3 weeks)	Donepezil transdermal delivery system (TDS)
13. Hussian et al. [19]	Case report	2012	Female, 84	Non-squamous erythematous papules, round lesions and vesicles on the trunk, arms, and legs—linear Ig A disease	4 weeks	Donepezil
14. Kortteenniemi et al. [20]	Case report	2019	Females—18 years old and	Skin erythema and nodular itchy lesion	After 2 and 6 days	Transcranial direct current stimulation
			Female—19 years old	Non-itchy and non-lumpy lesion	After 2 days	
15. Lu and Lam [21]	Case report	2019	1 male and 2 females—mean age 69 years old	Mild redness of the skin	Right after the first session	Transcranial direct current stimulation (cathodal electrode)
				Skin burns	The fourth or fifth session	
16. Frank et al. [22]	Case report	2010	3 patients	Mild skin burns	After the fourth session	Transcranial direct current stimulation (anodal electrode)
17. Maas et al. [23]	Case report	2021	Male—47 years old	2 targeted areas of skin burned in the tattoo below the electrode’s corners	After the tenth session	Transcranial direct current stimulation (cathodal electrode)
18. Wang et al. [24]	Case report	2015	Male—25 years old	Skin burns on frontal region	After a single session	Transcranial direct current stimulation (cathodal electrode)

**Table 2:** The most frequent skin lesions.

Method Implicated	Most Frequent Skin Lesions	Studies
Rivastigmine	Itchy rashes	[7,8,9,10,11,12,13,14,15]
Donepezil	Non-itchy lesions (papules, vesicles, skin irritation)	[18,19]
Memantine	Erythematous eruptions	[16,17]
Transcranial direct current stimulation	Skin burns	[20,21,22,23,24]

## Abbreviations

The following abbreviations are used in this manuscript:

WADA	World Anti-Doping Agency
TDCS	Transcranial direct current stimulation
